# Enhanced Uridine Bioavailability Following Administration of a Triacetyluridine-Rich Nutritional Supplement

**DOI:** 10.1371/journal.pone.0014709

**Published:** 2011-02-17

**Authors:** Melissa E. Weinberg, Mark C. Roman, Peyton Jacob, Michael Wen, Polly Cheung, Ulrich A. Walker, Kathleen Mulligan, Morris Schambelan

**Affiliations:** 1 University of California San Francisco, San Francisco, California, United States of America; 2 Tampa Bay Analytical Research Inc., Largo, Florida, United States of America; 3 Department of Rheumatology, Basel University, Basel, Switzerland; Sun Yat-Sen University, China

## Abstract

**Background:**

Uridine is a therapy for hereditary orotic aciduria and is being investigated in other disorders caused by mitochondrial dysfunction, including toxicities resulting from treatment with nucleoside reverse transcriptase inhibitors in HIV. Historically, the use of uridine as a therapeutic agent has been limited by poor bioavailability. A food supplement containing nucleosides, NucleomaxX®, has been reported to raise plasma uridine to supraphysiologic levels.

**Methodology/Principal Findings:**

Single- and multi-dose PK studies following NucleomaxX® were compared to single-dose PK studies of equimolar doses of pure uridine in healthy human volunteers. Product analysis documented that more than 90% of the nucleoside component of NucleomaxX® is in the form of triacetyluridine (TAU). Single and repeated dosing with NucleomaxX® resulted in peak plasma uridine concentrations 1–2 hours later of 150.9±39.3 µM and 161.4±31.5 µM, respectively, levels known to ameliorate mitochondrial toxicity *in vitro*. C_max_ and AUC were four-fold higher after a single dose of NucleomaxX® than after uridine. No adverse effects of either treatment were observed.

**Conclusions/Significance:**

NucleomaxX®, containing predominantly TAU, has significantly greater bioavailability than pure uridine in human subjects and may be useful in the management of mitochondrial toxicity.

## Introduction

Uridine, a pyrimidine nucleoside that plays an essential role in the synthesis of RNA and other key physiologic processes [1], has been used as a treatment for patients with hereditary orotic aciduria for more than four decades. The therapeutic potential of uridine has also been assessed in a diverse group of clinical disorders including cystic fibrosis, liver dysfunction, chemotherapy toxicity, pervasive developmental delay, schizophrenia, epilepsy, and diabetes-induced peripheral neuropathy [2]; and, recently, as a treatment for mitochondrial dysfunction associated with treatment with nucleoside reverse transcriptase inhibitors in patients with HIV infection [3]–[6].

Circulating levels of uridine are tightly regulated, in the range of 3–8 µM, both by the liver, which synthesizes and degrades uridine, and by erythrocytes that store UDP-glucose, which may be catabolized to provide glucose and uridine [2]. Uridine is absorbed from the gastrointestinal tract via facilitated diffusion or specific uridine transporters [7]. Historically, the use of uridine as a therapeutic agent has been limited by poor bioavailability. In a pharmacokinetic (PK) study of orally-administered uridine, the maximum tolerated dose was 10–12 g/m^2^ for a single dose and 5 g/m^2^ for a multiple-dose regimen (every 6 hours for 3 days), resulting in peak serum uridine concentrations of 60–80 µM after a single dose and a steady-state serum uridine level of 50 µM after multiple doses [8]. The dose-limiting side effect was diarrhea.

Recently, administration of NucleomaxX®, a food supplement that contains approximately 6 g of nucleosides (the equivalent of about 3.5 g/m^2^ in an average-sized male), achieved higher peak uridine levels (100–150 µM) during a single dose PK study [9] than were achieved in prior studies using uridine. NucleomaxX® is apparently well-tolerated. Although these results suggest that the formulation of uridine in NucleomaxX® may have enhanced bioavailability, the differences in plasma uridine levels achieved in studies of uridine and those of Nucleomaxx® could also be explained by the fact that different analytic techniques and study designs were employed. We therefore performed an analysis of the nucleoside components of NucleomaxX® and compared the PK profiles achieved following equimolar doses of the pyrimidine nucleosides provided by NucleomaxX® to that of pure uridine in healthy human volunteers. In addition, because the optimal dosing regimen of NucleomaxX® is not known, we also performed multiple-dose PK studies using NucleomaxX® to determine whether repeated dosing had a cumulative effect on plasma uridine levels.

## Methods

### Ethics Statement

All studies were approved by the Clinical and Translational Sciences Institute Clinical Research Center (CRC) at San Francisco General Hospital Advisory Committee and the University of California San Francisco Institutional Review Board. All subjects provided written informed consent.

### Product Analysis

To determine the total nucleoside content in NucleomaxX®, the contents of 10 sachets, each containing 36 gm of NucleomaxX®, were combined, homogenized and passed through a 60 mesh sieve. Nucleosides were extracted in aqueous solution containing ascorbic acid and quantified by reversed-phase high performance liquid chromatography (HPLC) with ultraviolet (UV) detection. Laboratory validation was performed to determine the repeatability, accuracy, selectivity, ruggedness and linearity of the method.

To determine the repeatability precision, four replicate test preparations of the NucleomaxX® product were prepared and analyzed on three separate days, for a total of 12 replicate preparations. The within-day, between-day, and total repeatability precision of the uridine and TAU content were calculated from the results. Accuracy was determined by spiking a placebo (a mixture of casein and sucrose) with uridine and TAU at three different concentrations in triplicate and determining the recovery. In addition, NucleomaxX® product was spiked with TAU and uridine in triplicate, and the recovery of each was determined.

The identities of the uridine and TAU in the NucleomaxX® material were confirmed by liquid chromatography-mass spectrometry/mass spectrometry (LC-MS/MS) on two separate systems. The primary study was conducted on a Waters 2695 solvent delivery system, Waters 2996 photodiode array detector, and Waters/Micromass Quattro Micro LC/MS/MS system. Chromatography was performed using gradient elution with mobile phases of 0.1% formic acid in water and methanol on a Sunfire 3.5 µm, 2.1×150 mm column and a mobile phase flow rate of 0.25 mL/min. Spray chamber conditions were: electron spray ionization (ESI) positive mode, cone voltage 35 KV, source temperature 120°C, desolvation temperature 300°C, and cone gas flow rate 50 L/min.

A secondary study to confirm TAU identity in NucleomaxX® was performed on a Varian 1200L HPLC-MS/MS with ESI source operated in positive mode using a C18 column and similar mobile phase conditions as the primary study.

### Pharmacokinetic studies of NucleomaxX®

Single- and multiple-dose PK studies of NucleomaxX®, supplied by PharmaNord (Spanga, Sweden), were performed in eight healthy volunteers (four men, four women) who were hospitalized in the CRC at San Francisco General Hospital for 7 days and fed a constant diet. The ingredients and nutritional composition of NucleomaxX® were provided by the manufacturer (Tables S1 and S2). On Day 1, one sachet (36 g) of NucleomaxX® was mixed with 300 mL of liquid and was administered as a single dose. All blood samples were collected on ethylene diamine tetraacetic acid. For the single-dose PK studies, plasma uridine levels were measured just prior to and 0.5, 1, 1.5, 2, 2.5, 3, 4, 6, 8, 12, 16, 20, and 24 hours following administration of NucleomaxX®. On Days 2–6, subjects were dosed with a single sachet every 8 hours, and uridine was measured 1, 2, 4, and 8 hours after each dose. On Day 7, the full-scale single-dose PK study was repeated.

### Pharmacokinetic studies of pure uridine

Single-dose PK studies using pure uridine (RG2417), supplied by Repligen Corporation (Waltham, MA), were also performed using a protocol identical to that described above in eight healthy volunteers (four men, four women) who were hospitalized overnight in the CRC. Based on the results of the independent product analysis described above, we administered RG2417, dissolved in water, in an amount designed to provide an equimolar amount of total nucleosides to that contained in a single sachet of NucleomaxX® (4.15 gm RG2417 [Repligen, Inc., Waltham, MA], containing 1.70×10^−2^ mol of uridine). The identical protocol used for the single-dose PK study of NucleomaxX® was employed.

### Plasma uridine assays

40 mM of ^13^C_9_ uridine (Spectra Stable Isotopes, Columbia, MD) as an internal standard was added to 50 µL of plasma, which was subsequently denatured with 1 mL of 2-propanol. Supernatants were isolated after 5 minutes of centrifugation to pellet precipitates (5000 rpm, 4°C). Samples were then evaporated to dryness using a Speedvac for 60 minutes, resuspended in 1 mL of 10 mM ammonium formate, purified through a 0.02 µM Whatman anotop syringe filter, and frozen at −20°C until LC-MS analyses were performed.

Samples were introduced on a SpectraSystem P4000 HPLC system equipped with an AS3000 SpectraSystem autosampler (Thermo Electron, San Jose, CA, USA). 50 µL of sample containing uridine and internal standard were chromatographed on a 4.0 mm ×150 mm Supelco Discovery HSF5 5 micron column fitted with an HSF5 guard column, 4.0 mm ×20 mm (Supelco, Bellefonte, Pennsylvania, USA). The column was first conditioned by a mobile phase of 10 mM ammonium formate in HPLC water at a flow rate of 0.7 mL/min for 4 minutes. Then the isocratic LC method used a mobile phase of 10 mM ammonium formate in methanol at a flow rate of 0.7 mL/min for 3 minutes. The column was reconditioned by 10 mM ammonium formate in HPLC water at a flow rate of 0.7 mL/min for 4.9 minutes before the next injection. The LC effluent from the sample injections was directed to the APCI source on the TSQ 7000.

Selected Reaction Monitoring (SRM) on the Thermo Finnigan TSQ 7000 (Thermo Electron, San Jose, CA, USA) was used for detection of both uridine and ^13^C_9_ uridine as internal standard. Mass spectrometer parameters for the LC/MS/MS assay were as follows: Ion source atmospheric pressure chemical ionization; ion polarity positive; sheath gas pressure 60 psi; capillary temperature 250°C; vaporizer temperature 450°C; corona discharge current (uA) 4.0. For uridine, SRM parameters on the TSQ 7000 were: SRM transition 245.1→113.0; collision energy 21; scan time 0.1. For ^13^C_9_ uridine, SRM parameters on the TSQ 7000 were: SRM transition 254.1→ 117.0; collision energy 19; scan time 0.1.

### Pharmacokinetic and statistical analyses

For PK analyses, baseline levels of plasma uridine measured prior to administration of either NucleomaxX® or pure uridine were subtracted from all subsequent values in order to account for endogenous levels of uridine. The Day 1 baseline uridine level was subtracted from values in the Day 7 analysis, rather than Day 7 baseline uridine levels, which were higher due to prior exogenous administration of NucleomaxX®. PK parameters were determined using a noncompartmental model and the “best fit” lambda z calculation method (WinNonlin, Pharsight Corp., Mountain View, CA). AUC was calculated using the trapezoidal method. In order to explain the observed differences in results obtained in the men and women, analyses were also performed adjusting for lean body mass as determined by dual energy X-ray absorptiometry (Hologics Discovery Wi, Waltham, MA). PK parameters obtained for NucleomaxX® on Days 1 and 7 were compared using a paired t-test. Student's t-test was used to compare the Day 1 PK parameters of NucleomaxX® and uridine (RG2417).

## Results

### Product Analysis

**Figure 1:**
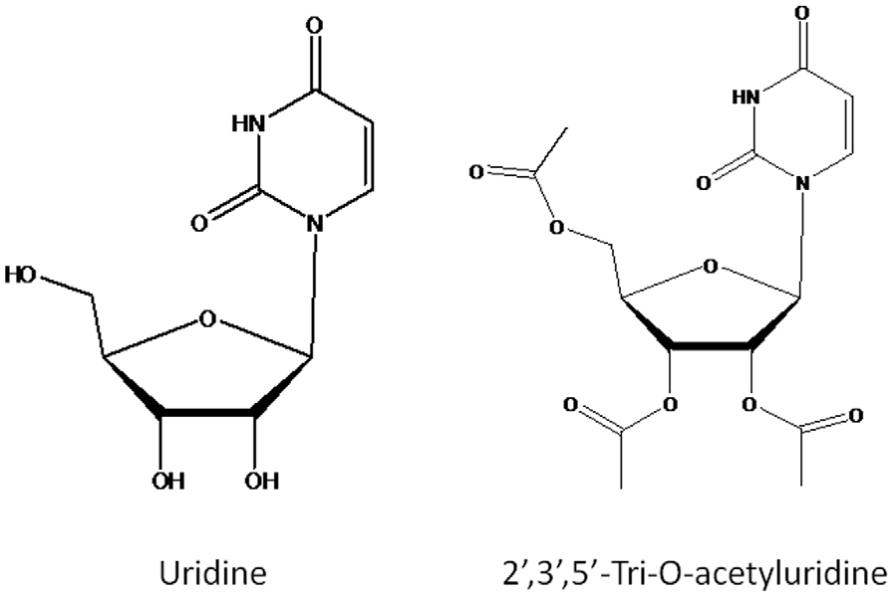
Structures of Uridine and 2′,3′,5′-Tri-O-acetyluridine (TAU).

Each sachet of NucleomaxX® contained 0.58 g (1.61% by weight) uridine and 5.4 g (15.0%) 2′,3′,5′-tri-O-acetyluridine (Figure 1) as determined by HPLC-UV. The analytical method for the determination of uridine and TAU was validated according to Association of Official Analytical Chemists International guidelines for single-laboratory validation for accuracy, precision, selectivity, linearity/range, ruggedness, and limits of detection and quantitation. The identity of TAU was verified by HPLC with tandem mass spectroscopy. Uridine and TAU recoveries from NucleomaxX® were 98.2% –104.8%, and 98.3–101.5%, respectively, and repeatability precision was 2.43% relative standard deviation (RSD) for uridine, and 2.00% RSD for TAU. When the uridine and TAU content are combined, we calculated that each sachet of NucleomaxX® contained the equivalent of 1.70×10^−2^ mol of total uridine.

### Subjects

Separate PK studies were performed using NucleomaxX® and pure uridine. There were no significant differences in baseline characteristics between the men who participated in the two studies (Table 1). Among the women, those participating in the NucleomaxX® study had a slightly lower lean body mass (36.6 vs. 39.8 kg, p = 0.03) than in the uridine group. No adverse effects of either NucleomaxX® or pure uridine were reported by the study volunteers.

**Table 1:** Subject Characteristics in the PK studies of NucleomaxX® and uridine (RG2417).

Men	NucleomaxX® (n = 4)	Uridine(n = 4)
Age (years)	39.5 (12.8)	30.0 (7.9)
Weight (kg)	80.8 (7.3)	87.2 (7.8)
BMI (kg/m^2^)	26.1 (2.7)	26.0 (3.1)
Total fat (kg)	19.4 (5.3)	17.4 (4.7)
Total lean (kg)	57.3 (4.2)	65.4 (8.3)
Percent fat mass (%)	24.2 (5.1)	20.3 (5.9)

Women	(n = 4)	(n = 4)
Age (years)	30.3 (9.5)	31.3 (12.0)
Weight (kg)	60.1 (6.6)	60.0 (11.0)
BMI (kg/m^2^)	24.0 (3.6)	22.0 (2.4)
Total fat (kg)	20.5 (4.2)	17.1 (5.9)
Total lean (kg)	36.6 (3.0)	39.8 (5.4)*
Percent fat mass (%)	34.3 (4.1)	28.3 (4.9)

*p = 0.03 vs women studied with NucleomaxX®.

Data are presented as mean (SD).

### Uridine pharmacokinetics following administration of NucleomaxX®

**Figure 2:**
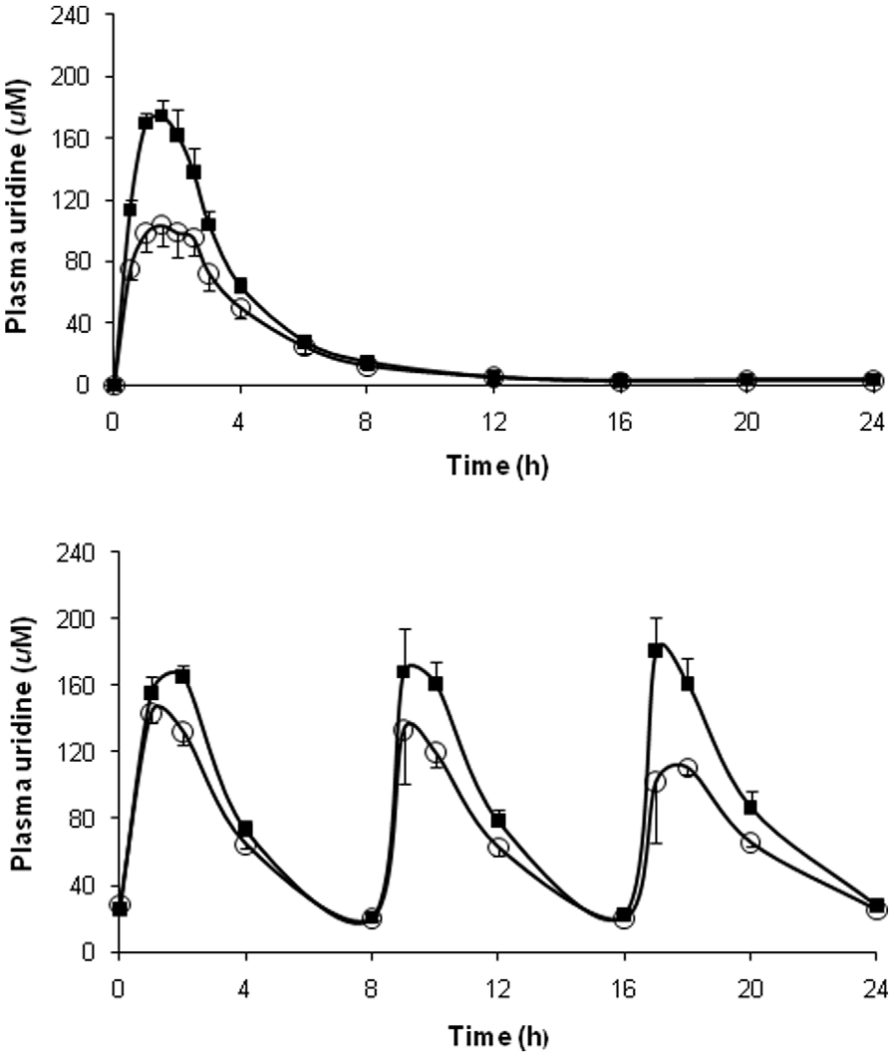
Single- and multi-dose PK profiles of uridine. (a) PK profile of uridine following a single-dose of NucleomaxX® in all subjects. The solid squares indicate female subjects, and the open circles indicate male subjects. (b) PK profile of uridine following three doses of NucleomaxX® in all subjects. The solid squares indicate female subjects, and the open circles indicate male subjects.

**Figure 3:**
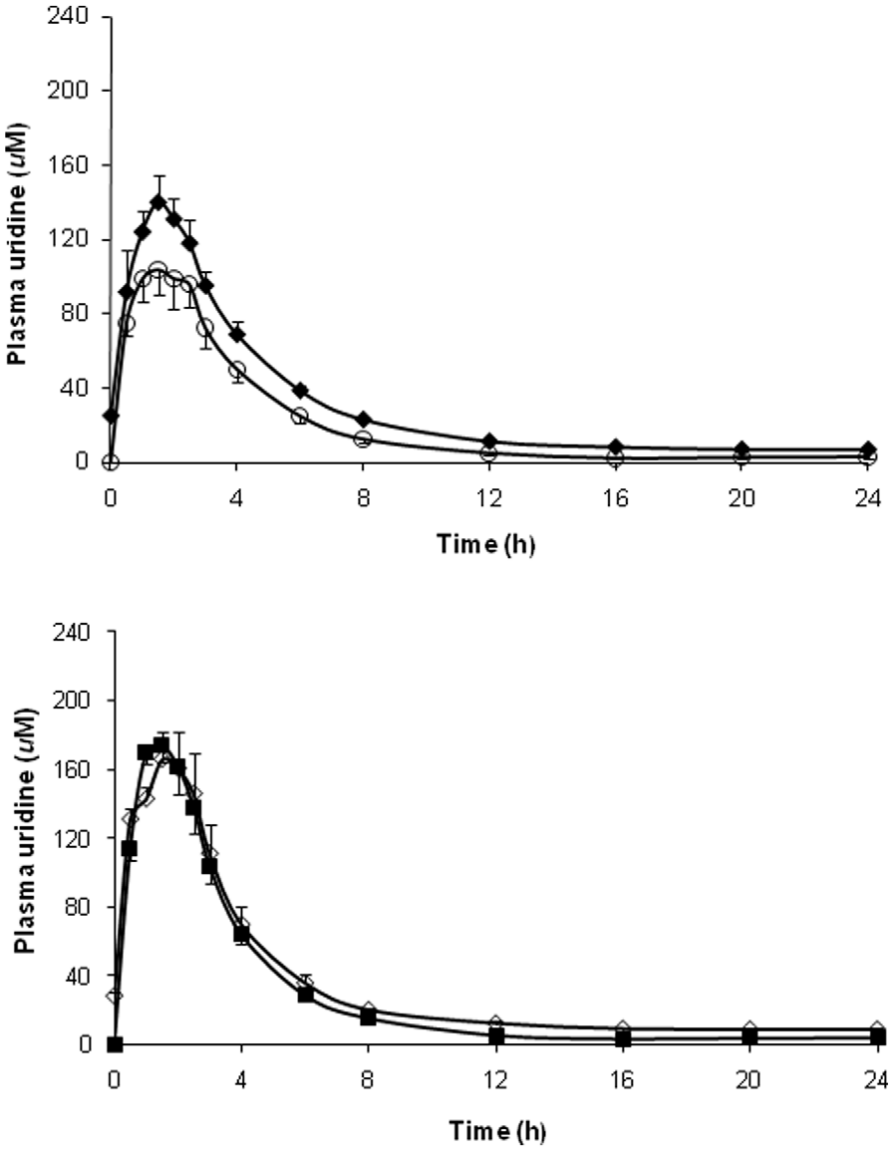
Comparison of PK profiles of uridine following NucleomaxX® administration on Day 1 and Day 7. (a) The open circles indicate day 1 (single-dose), and the solid diamonds indicate day 7 (cumulative-dosing) in men. (b) The solid squares indicate day 1 (single-dose), and the open diamonds indicate day 7 (cumulative-dosing) in women.

At baseline (t = 0 on Day 1), mean plasma uridine level was 4.7 uM, consistent with known physiologic concentrations of this nucleoside [2]. Plasma uridine levels increased to similar maximal concentrations (>150 µM) 1–2 hours after dosing (Table 2) on both Day 1 and Day 7. The half-life of uridine after cumulative dosing was longer than that observed after a single dose (5.3±1.4 vs. 3.4±0.8 hours, p = 0.01). Plasma uridine levels at t = 0 as well as mean uridine area under the curve (AUC) and AUC_∞_ were greater on Day 7 than on Day 1, but the latter could be attributed to the higher baseline level of uridine on Day 7. With both the single- and multi-dose NucleomaxX® regimens, women achieved higher uridine concentrations than men (Figure 2). When plasma uridine levels were adjusted for body weight or lean body mass, the differences between men and women were no longer evident. Among the men, plasma uridine levels were higher on Day 7 compared with Day 1 at all time points (Figure 3a), but among the women, there were no significant differences in the PK profiles on days 1 and 7 (Figure 3b).

**Table 2:** PK parameters for NucleomaxX® (Day 1 &7) and an equimolar amount of pure uridine (RG2417).

	NucleomaxX®			Uridine	
	Day 1	Day 7	p-value	Day 1	p-value*
C_0_ (µM)	4.7 (2.1)	31.1 (4.9)	<0.0001	4.1 (1.4)	NS
C_max_ (µM)	150.9 (39.3)	161.4 (31.5)	NS	36.1 (11.3)	<0.0001
t_max_ (h)	1.3 (0.5)	1.5 (0.5)	NS	1.9 (0.9)	NS
t_1/2_ (h)	3.4 (0.8)	5.3 (1.4)	0.01	4.6 (1.2)	0.05
AUC (µM/h)	602.5 (143.3)	786.4 (115.9)	0.002	143.0 (57.7)	<0.0001
AUC_inf_ (µM/h)	620.8 (140.5)	843.6 (120.9)	0.0003	164.1 (59.4)	<0.0001
AUC_inf_ adjusted for baseline (µM/h)	599.5 (140.0)	606.2 (102.8)	NS	135.4 (64.5)	<0.0001

*vs. Day 1 of NucleomaxX®.

Data are presented as mean (SD).

### Uridine pharmacokinetics following administration of pure uridine (RG2417)

**Figure 4:**
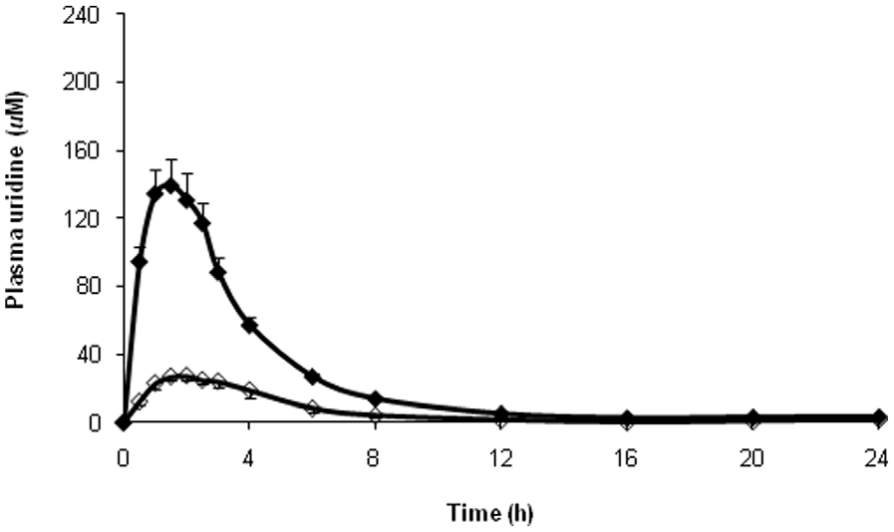
PK profile of uridine after single doses of NucleomaxX® and pure uridine in all subjects. An equimolar amount of NucleomaxX® and pure uridine (RG2417) was used. The solid diamonds indicate NucleomaxX®, and the open diamonds indicate pure uridine.

Mean plasma uridine level prior to drug administration (t = 0) was similar in both PK studies (4.1±1.4 vs. 4.7±2.1 uM, for the uridine and NucleomaxX® studies, respectively; p = NS). When the single-dose uridine PK profile of NucleomaxX® (containing predominantly TAU) was compared with that of pure uridine using the same PK protocol and laboratory assay, four-fold greater bioavailability was observed with NucleomaxX®. The maximal uridine concentration, AUC, and AUC_∞_ were significantly higher in both men and women (Table 2 and Figure 4). The mean maximal concentration of uridine after administration of pure uridine was 36.1±11.3 µM compared with 150.9±39.3 µM after NucleomaxX® (p<0.0001), also occurring 1–2 hours after dosing. The half-life for pure uridine was longer than that for NucleomaxX® (4.6±1.2 vs. 3.4±0.8 hours, p = 0.05).

## Discussion

Both single and repeated dosing with NucleomaxX® resulted in peak plasma uridine concentrations >150 µM, levels known to ameliorate mitochondrial toxicity *in vitro*, without any adverse effects. Women achieved higher uridine concentrations than men, likely attributable to their smaller body size. The men demonstrated higher uridine levels after six days of cumulative administration of NucleomaxX® compared with Day 1, but the women showed no further rises in uridine concentration. Given the small number of subjects, no definitive conclusions can be made regarding these apparent gender differences in the uridine bioavailability of NucleomaxX®, but these results invite further study. C_max_ and AUC were four-fold higher after administration of a single dose of NucleomaxX® than that found after an equimolar amount of pure uridine (RG2417). The finding that >90% of the nucleoside component of NucleomaxX® is in the form of TAU, rather than uridine, suggests that TAU has much greater bioavailability than pure uridine.

To our knowledge, this is the first study to demonstrate enhanced bioavailibility of TAU compared with uridine in humans. In mice, the relative bioavailability of plasma uridine from orally administered TAU was 7-fold greater than that achieved by an equimolar amount of oral uridine [10]. The increased bioavailability of TAU is likely due to its molecular structure, since the additional acetyl groups result in greater lipophilicity and, therefore, enhanced gastrointestinal tract absorption [11]. Unlike uridine, TAU does not require a pyrimidine transporter for absorption. In addition, TAU is resistant to catabolism by uridine phosphorylase [12]. It is converted into uridine by intestinal and plasma esterases cleaving the acetyl groups, allowing sustained tissue delivery of uridine [11].

Our findings in the single-dose PK study of NucleomaxX® are consistent with those previously reported by Venhoff et al. [9]. Even though subjects were studied and samples were analyzed in different laboratories, key results were remarkably similar. In our study and the study by Venhoff et al., baseline uridine concentrations were 4.7 and 5.6 µM and maximal uridine concentrations were 150.9 and 152.0 µM, respectively. Time to maximal concentration was 1.3 h in both studies. We extend these previous findings to include pharmacokinetics of uridine after the administration of multiple doses of NucleomaxX® (three times per day) and after cumulative dosing for six days. The rapid, dramatic rises in plasma uridine observed after administration of NucleomaxX® contrast with those seen with equimolar amounts of pure uridine, highlighting the potentially benefical pharmacokinetics of this dietary supplement.

Positive effects of uridine on mitochondrial function and oxidative metabolism have been demonstrated in both cell culture and animal models. In mammalian cells artificially depleted of mtDNA (*rho* cells) [13] as well as in fibroblasts from humans with decreased levels of mtDNA (due to a nuclear genetic defect in respiratory-chain function) [14], uridine and pyruvate supplementation are required to maintain growth. Ehrlich ascites tumor cells grown in glucose-deprived medium supplemented with uridine increased oxygen uptake by 50% and reduced lactate production to 10% of that in control cells [15]. ATP production was unchanged, despite a reduction in glycolysis, due to an increase in oxidative pathways. Uridine also increased myocardial performance, glucose uptake and glycolysis, in addition to diminishing the disappearance of glycogen and adenine nucleotides from hypoxic rabbit hearts [16].

Mitochondrial dysfunction has been proposed as the unifying feature of NRTI-induced toxicity in patients with HIV infection. Because uridine supplementation may favor mtDNA synthesis by increasing the pyrimidine pool, it has been investigated as a potential therapy in this setting. Uridine reversed the toxic effects of zidovudine on colony formation in human bone marrow progenitor cells without impairing the antiretroviral effect of this agent [4]. Uridine was also found to prevent zalcitabine-induced toxicity in PC12 cells, a neuronal model used to study peripheral neuropathy [5]. In human HepG2-hepatocytes exposed to NRTIs, uridine normalized cell proliferation, lactate levels, and intracellular lipids by increasing mtDNA levels to about 65% of NRTI-unexposed control cells [6].

Published data on the effects of uridine on NRTI-induced toxicity in humans are limited. A single case report described a remarkable salutary effect of only four days of treatment with NucleomaxX®, ameliorating the mitochondrial toxicity caused by stavudine and leading to improvements in myalgias, liver and muscle enzymes, lactate levels, and steatosis, despite continuing treatment with stavudine [17]. Recently, a randomized, double-blind, placebo-controlled trial of NucleomaxX® for the treatment of HIV-associated lipoatrophy reported a significant increase in limb fat, intra-abdominal fat, and total body fat [18]. Our study, which demonstrates that the nucleoside content of NucleomaxX® is predominantly TAU, may explain why such remarkable clinical results have been obtained with NucleomaxX® as the source of uridine supplementation. However, the optimal therapeutic dose and plasma or intracellular uridine levels for treatment of NRTI-associated toxicity are not known.

Although it remains possible that another component of NucleomaxX® explains the enhanced bioavailability of uridine, we were unable to study pure TAU due to its lack of availability. Moreover, we felt that investigation of NucleomaxX® was more relevant to current clinical practice since it is already being used by HIV-infected individuals. Given the considerable interest among these patients in the use of complementary and alternative medicine [19], including food supplements, even though scientific evidence of their efficacy is scarce, there is clearly need for rigorous scientific studies investigating both the content and the mechanism of these supplements.

Mitochondrial dysfunction may also play a role in the abnormal glucose metabolism resulting from NRTI toxicity, and perhaps diabetes in non-HIV-infected individuals. The proposed mechanism entails reduced oxidative enzyme capacity, thereby increasing lipid accumulation in muscle and liver. These fatty acids and their metabolites inhibit insulin-stimulated glucose transport, subsequently leading to insulin resistance [20]. The ATP generated by oxidative phosphorylation in the mitochondria is also necessary for glucose sensing and exocytosis of insulin granules, leading to impaired insulin secretion in the β-cells of the pancreas [21].

If mitochondrial dysfunction is, indeed, the underlying cellular defect that explains abnormal glucose metabolism, agents that enhance mitochondrial function—such as uridine— may improve glucose homeostasis in this and other insulin-resistant states (e.g. type 2 diabetes, the metabolic syndrome, polycystic ovary syndrome, and nonalcoholic steatohepatitis). Our study found that administration of NucleomaxX®, containing predominantly TAU, was able to overcome prior issues of poor bioavailability with pure uridine in human subjects with no adverse effects. Given its ability to raise and sustain supraphysiologic uridine levels, TAU may be useful in the management of disorders previously treated with uridine.

## Supporting Information

Table S1 Ingredients of NucleomaxX®.
(0.03 MB DOC)

Table S2 Nutritional composition of NucleomaxX®.
(0.03 MB DOC)
