# Supplementary material for: Enhanced Uridine Bioavailability Following Administration of a Triacetyluridine-Rich Nutritional Supplement
Source: PLoS One. 2011 Feb 17;6(2):e14709. doi: 10.1371/journal.pone.0014709 (PMC3040752; doi:10.1371/journal.pone.0014709)
Supplement: Table S1 — Ingredients of NucleomaxX®. (0.03 MB DOC) [file pone.0014709.s001.doc]

| **Ingredient** | **Percentage (%)** |
| --- | --- |
| Sugar cane extract | 67 |
| Milk protein | 22 |
| Aroma | 11 |
| Sweeteners: |  |
| acesulfam-K | 0.1 |
| aspartame | 0.2 |
| sodium cyclamate | 0.1 |
| saccharin sodium | 0.1 |
| Total | 100 |
