# Supplementary material for: Enhanced Uridine Bioavailability Following Administration of a Triacetyluridine-Rich Nutritional Supplement
Source: PLoS One. 2011 Feb 17;6(2):e14709. doi: 10.1371/journal.pone.0014709 (PMC3040752; doi:10.1371/journal.pone.0014709)
Supplement: Table S2 — Nutritional composition of NucleomaxX®. (0.03 MB DOC) [file pone.0014709.s002.doc]

| **Nutrient** | **Weight (g/100 g NucleomaxX®)** |
| --- | --- |
| Protein | 27.7 |
| Fat | 2.83 |
| Carbohydrate | 63.7 |
| Ash | 3.31 |
| Water | 2.34 |
